# Supplementary material for: Correction: Connexin 43 plays an important role in the transformation of cholangiocytes with Clonochis sinensis excretory-secretory protein and N-nitrosodimethylamine
Source: PLoS Negl Trop Dis. 2019 Jun 28;13(6):e0007526. doi: 10.1371/journal.pntd.0007526 (PMC6599143; doi:10.1371/journal.pntd.0007526)
Supplement: S2 File — (PDF) [file pntd.0007526.s002.pdf]

PLOS | NEGLECTED TROPICAL DISEASES

RESEARCH ARTICLE

Connexin 43 plays an important role in the transformation of cholangiocytes with *Clonochis sinensis* excretory-secretory protein and *N*-nitrosodimethylamine

Eun-Min Kim[1], **Young Mee Bae**[2], **Min-Ho Choi**[2], **Sung-Tae Hong**[2]*

1 Department of Environmental Medical Biology and Arthropods of Medical Importance Resource Research Bank, Institute of Tropical Medicine, Yonsei University College of Medicine, Seoul, Republic of Korea, **2** Department of Parasitology and Tropical Medicine and Institute of Endemic Diseases, Seoul National University College of Medicine, Seoul, Republic of Korea

* hst@snu.ac.kr

Check for updates

OPEN ACCESS

Citation: Kim E-M, Bae YM, Choi M-H, Hong S-T (2019) Connexin 43 plays an important role in the transformation of cholangiocytes with *Clonochis sinensis* excretory-secretory protein and *N*-nitrosodimethylamine. PLoS Negl Trop Dis 13(4): e0006843. https://doi.org/10.1371/journal.pntd.0006843

Editor: John Pius Dalton, Queen's University Belfast, UNITED KINGDOM

Received: September 12, 2018

Accepted: December 11, 2018

Published: April 3, 2019

Copyright: © 2019 Kim et al. This is an open access article distributed under the terms of the Creative Commons Attribution License, which permits unrestricted use, distribution, and reproduction in any medium, provided the original author and source are credited.

Data Availability Statement: All relevant data are within the manuscript and its Supporting Information files.

Funding: The present work was partially supported by the Education and Research Encouraging Fund of the Seoul National University Hospital, 2017. The funders had no role in study design, data collection and analysis, decision to publish, or preparation of the manuscript

Abstract

Background

Clonorchis sinensis is a group I bio-carcinogen responsible for cholangiocarcinoma (CHCA) in humans. However, the mechanism by which *C. sinensis* promotes carcinogenesis is unclear.

Methodology

Using the human cholangiocyte line H69, we investigated cell proliferation and gap junction protein expression after stimulation with the hepatotoxin *N*-nitrosodimethylamine (NDMA) and/or excretory-secretory products (ESP) of C. *sinensis*, which induce inflammation. NDMA and ESP treatment increased proliferation by 146% and the proportion of cells in the G2/M phase by 37%. Moreover, the expression of the cell proliferation-related proteins E2F1, Ki-67, and cancer related protein cytokeratin 19 and Cox-2 increased in response to combined treatment with NDMA and ESP. The gap-junction proteins connexin (Cx) 43 and Cx26 increased. In contrast, Cx32 expression decreased in cells treated with NDMA and ESP. Silencing of Cx43 reduced cell proliferation and significantly suppressed Cx26 and Cox-2 expression.

Conclusions

These results suggest that Cx43 is an important factor in CHCA induced by *C. sinensis* ESP and NDMA and further investigations targeting this pathway may allow prevention of this deadly disease.

Competing interests: The authors of this manuscript have no financial conflicts of interest.

Author summary

Clonorchis sinensis, a human fluke, resides in the liver of humans and is commonly found in the common bile duct and gall bladder. This parasite is the main cause of cholangiocarcinoma, also called bile duct cancer, in humans. Of note, the excretory-secretory products (ESP) of *C. sinensis* are known to cause inflammation in the biliary epithelium, which may ultimately result in neoplasms via production of reactive oxygen species and subsequent DNA damage. Together with *N*-nitrosodimethylamine (NDMA), a potent hepatotoxin that can cause fibrosis and tumors in the liver, ESP led to an increase in the growth and proliferation of cholangiocytes. Our results showed that examination of changes in the expression of gap junction proteins, which are related to tumorigenesis, showed that connexin 43 was upregulated with ESPs from *C. sinensis* and NDMA. Together, our results suggest that exposure to *C. sinensis*, in addition to low levels of carcinogen could promote carcinogenesis in the bile duct epithelium via uncontrolled cell-to-cell communication. Moreover, silencing of Cx43 reduced cancer related protein. Therefore, Cx 43 can serve as a potential target for developing a therapeutic strategy for the treatment of cholangiocarcinoma in humans.

Introduction

Clonorchis sinensis is a human liver fluke that induces cholangiocarcinoma (CHCA) in humans [1]. Clonorchiasis has been endemic in Asia for a long time, especially among residents who live along rivers and consume raw freshwater fish [2].

The mechanism by which *C. sinensis* induces CHCA is not well understood, but chronic hepatobiliary damage, a precursor to CHCA, in clonorchiasis is a multi-factorial outcome of the mechanical and biochemical irritation of the biliary epithelium by flukes via their suckers, metabolites, and excretory-secretory products (ESP) [3]. Local inflammation and the systemic immune response in the host [4–7] produce reactive oxygen species and reactive nitrogen compounds, which may cause DNA damage, leading to neoplasms [8, 9].

N-nitrosodimethylamine (NDMA) is a potent hepatotoxin that can cause fibrosis and tumors in the liver of rats via the activation of CYP450 enzymes [10] and hamsters infected with *C. sinensis* are at a greater risk of developing NDMA-induced or inflammation-mediated CHCA than uninfected hamsters [11, 12]. Previously, we reported that exposure to NDMA and the ESP of *C. sinensis* increases HEK293T cell proliferation and the proportion of cells in the G2/M phase [3].

CHCA is potentially caused by increased levels of proinflammatory cytokines and nuclear factor kappa B (NFκB), which regulate the activities of cyclooxygenase-2 (Cox-2) and inducible nitric oxide synthase, and disturb the homeostasis of oxidants/anti-oxidants and DNA repair enzymes [13]. ESPs of *C. sinensis* induce pro-inflammatory responses *in vitro* by the upregulation of TLR4 and its downstream transduction signals, including MyD88-dependent IκB-α degradation and NFκB activation [14, 15]. NFκB may also influence the production of Cx43, a gap-junction protein, in liver cirrhosis [16, 17].

Gap junctions are clusters of transmembrane channels on the cell membrane that permit direct intercellular exchange of ions, secondary messengers, and small signaling molecules influencing cell growth, differentiation, and cancerous changes [17–21]. Among gap junction proteins, Cx43 is involved in almost all steps of the inflammatory response of cells, cytokine production, and inflammatory cell migration [17, 20, 22]. The substantial role of Cx43 in

carcinogenesis is highlighted by the fact that high levels of Cx-43 expression increase the invasion of breast tumor cells and promote tumors in melanoma [22].

Alterations of Cx expression have been reported in cancer [21, 23]. In hepatocellular carcinoma (HCC), for instance, reduced Cx32 expression is accompanied by increased expression of Cx43, which promotes HCC via cell-to-cell communication [16, 20, 23]. Fujimoto et al. [23] have shown that Cx32 has a suppressive effect in metastatic renal cell carcinoma. However, the role of connexins in cancer is still controversial [23], and the influence of gap junctions in CHCA caused by *C. sinensis* has not yet been examined.

In this study, to determine the mechanisms underlying the carcinogenic effects of ESP from *C. sinensis*, we investigated changes in cell proliferation, proinflammatory molecules, and connexin production in cholangiocytes (H69 cell line) exposed to ESP from *C. sinensis* and the carcinogen NDMA. We found that the silencing of Cx43 reduced ESP- and NDMA-induced cell proliferation and the expression of Cox-2.

Methods

Animals

The animal experimental protocol was approved and reviewed by the Institutional Animal Care and Use Committee (IACUC) of Seoul National University Health System, Seoul, Korea (approval no. SNU-060511-1) and followed the National Institutes of Health (NIH) guideline for the care and use of laboratory animals (NIH publication no. 85–23, 1985, revised 1996). The facility is accredited by the Ministry of Food and Drug Administration and by the Ministry of Education, Science and Technology (LNL08-402) as an animal experiment facility. Male Sprague–Dawley rats at 6 weeks of age were purchased from Koatech Co. (Seoul, Korea) and housed in an ABL-2 animal facility at Seoul National University College of Medicine. All rats were bred in filter cages under positive pressure according to institutionally approved guidelines.

Recovery of metacercariae of *C. sinensis*

Pseudorasbora parva, the second intermediate host of *C. sinensis*, which was naturally infected *with C. sinensis*, was purchased from Sancheong-*gun*, *Gyeong*sangnam-do, Republic of Korea. This is an endemic area for clonorchiasis, which we have previously described [2]. Metacercariae of *C. sinensis* were collected by digestion of fish with pepsin-HCl (0.6%) artificial gastric juice for 1 h at 37˚ C, then filtering the digestion solution through a 200㎛ stainless steel filter. Finally, and *C. sinensis* metacercariae were isolated under stereomicroscopic identification.

Infection of experimental animals with *C. sinensis* and collection of ESP

Sprague–Dawley rats were individually infected orally with 50 metacercariae of *C. sinensis*. Eight weeks post-infection, adult worms were collected from b*ile ducts a*nd washed several times with phosphate-buffered saline (PBS). The freshly isolated worms were then incubated in sterile PBS containing antibiotics for 24 h in an atmosphere of 5% CO_2 at 37˚C. After incubation, the medium was centrifuged for 10 min at 800 rpm to remove the worms and debris. The supernatant was then further centrifuged for 10 min at 3000 rpm and filtered with a syringe-driven 0.45-μm filter unit. The amount of protein in each extract was measured using the Bradford assay (Thermo, Rockford, IL, USA). The concentration of endotoxin (LPS) was measured using an LAL QCL-1000 Kit (LONZA, Switzerland), and as a result, the LPS

contained in 10 μg/mL of ESP was confirmed to be less than 0.001 (EU). Therefore, there is no effect of LPS on the results of this paper.

Cell culture

SV40-transformed human cholangiocytes (H69) from Dr. Dae-Gon Kim of Chonbuk National University for providing were divided into four treatment conditions and cultured for more than 180 days; the medium was replaced every 72 h. The cells were then treated as follows: control, cultured in plain medium; 100 ng/mL NDMA, cultured in medium containing 100 ng/mL NDMA; ESP, cultured in medium containing 10 μg/mL ESP; and NDMA + ESP, cultured in medium containing 10 μg/mL NDMA and 100 ng/mL ESP. H69 cells were cultured in Dulbecco's modified Eagle's medium (DMEM; Gibco, Carlsbad, CA, USA) and DMEM/F12 supplemented with 10% heat-inactivated fetal bovine serum (FBS; Gibco), 2 mM L-glutamine, 100 μg/mL penicillin, 0.243 mg/mL adenine (Sigma, St. Louis, MO, USA; A68626), 5 μg/mL insulin (Sigma; I6634), 10 μg/mL epinephrine (Sigma; E4250), 5 μg/mL Triiodonine-transferrin (Sigma; T8158), 30 ng/mL epidermal growth factor (R&D Systems, Minneapolis, MN, USA; 236-EG), 1.1 μM hydrocortisone, and 100 U/mL streptomycin at 37˚C in a humidified atmosphere of 5% CO_2.

Cell proliferation assay

The PrestoBlue cell viability reagent was utilized to evaluate cell proliferation. For each assay, cells were seeded at a density of 5×10^3 cells/well on 96-well plates. After 24 h of incubation, the medium was replaced with 2% FBS-DMEM without phenol red. The cells were then incubated in the presence of PBS (vehicle) or with 100 ng/mL NDMA with or without 10 μg/mL ESP for another 72 h. The PrestoBlue cell viability reagent (1 mg/mL) was dissolved in warm medium, and 1.25 mM phenazine methosulfate (PMS) was prepared in PBS. Following incubation for the indicated periods, 50 μL of the PrestoBlue cell viability reagent was added to each well. The plates were then incubated for 1 h. The conversion of PrestoBlue cell viability reagent was quantified by measuring the absorbance at 570 and 600 nm using a microtiter plate reader.

Cell cycle analysis

For the cell cycle analysis, H69 cells were plated in six-well culture plates at 2×10^5 cells/well in 2 mL of DMEM containing 10% FBS. They were then treated with 100 ng/mL NDMA with or without 10 μg/mL ESP for 72 h and stained with propidium iodide (PI). The PI-stained cells were analyzed using a FACSCalibur multicolor flow cytometer (Becton-Dickinson, Franklin lakes, NJ, USA), and data were analyzed using CellQuest software (Becton-Dickinson).

Western blotting

For western blots, cells were lysed using 1% Nonidet P-40 in a buffer containing 150 mM NaCl, 10 mM NaF, 1 mM PMSF, 200 μM Na_3VO_4, and 50 mM HEPES, pH 7.4. Equal amounts of protein were separated by 8% and 10% sodium dodecyl sulfate-polyacrylamide gel electrophoresis (SDS-PAGE) and transferred to polyvinylidene fluoride (PVDF) membranes (Immobilon; Millipore, Billerica, MA, USA). The membranes were then probed with antibodies against E2F1, Ki-67, Ck19, Cox-2, connexin 43, connexin 32, connexin 26, and calnexin. The primary antibodies were detected using goat anti-rabbit or rabbit anti-mouse secondary antibodies conjugated with HRP and visualized using an enhanced chemiluminescence kit (ECL; Amersham Pharmacia Biotech, North Massapequa, NY, USA). The western blotting results were obtained by a densitometric analysis using ImageJ (NIH, Bethesda, MD, USA).

Antibodies

Polyclonal or monoclonal antibodies were used to detect the expression of cell-proliferation related proteins, including anti-E2F1 (sc-193; Santa Cruz Biotechnology, Santa Cruz, CA, USA) and anti-Ki-67 (SP6; Abcam, Cambridge, MA, USA). The other antibodies included anti-Cox-2 (c-9897; Cayman Chemicals, Ann Arbor, MI, USA) and anti-cytokeratin-19 (Ab53119; Abcam) as cancer-related makers, and anti-connexin 26 (138100; Invitrogen, Carlsbad, CA, USA), anti-connexin 32 (358900; Invitrogen), and anti-connexin 43 (138300; Invitrogen). An antibody against calnexin (BD 610523) used as a control was purchased from Transduction Laboratories (BD Biosciences, San Jose, CA, USA) and used at a 1:1,000 dilution. Anti-mouse, anti-rabbit, and anti-goat IgG antisera conjugated with horseradish peroxidase (HRP) were purchased from DAKO (Glostrup, Denmark).

Confocal microscopy

Cells were washed with cold PBS three times and fixed with 2% paraformaldehyde in PBS for 30 min. Permeabilization was performed by treating the cells with 0.2% (w/v) Triton X-100 in PBS for 5 min and then blocking with 0.5% BSA in PBS for 1 h. After blocking, the cells were incubated with primary antibodies against connexin 26, 32, and 43 (Invitrogen) diluted in BSA-PBS at 25˚C for 2 h and then incubated in secondary antibodies diluted in BSA-PBS at room temperature for 30 min. After washing with 1× PBS, the cells were stained with DAPI and observed under a confocal laser scanning microscope (LSM PASCAL; Carl Zeiss, Jena, Germany).

Cx43 silencing with siRNA

Three selected human Cx43-siRNAs (TriFECTa Kit disRNA Duplex, IDT, San Jose, CA, USA), with negative and positive controls, and specific siRNA targeting connexin 43 (NM-00165) (Table 1) were purchased from IDT. The transfection experiments were performed using the TransIT-TKO Kit (Mirus, Madison, WI, USA) following the manufacturer's instructions. Briefly, a 25 nM siRNA solution was mixed with 10 μL of TransIT-TKO and added to the wells of a 6-well plate containing 2×10^5 H69 cells for 72 h. Cells were then treated with NDMA, ESP of *C. sinensis*, or the combination for 72 h. Medium and cells (rinsed 2 times with ice-cold PBS) were harvested 3 days later. The efficiency of transfection was assessed by measuring the expression of gap junction proteins (connexin 26, connexin 32, and connexin 43) by real-time PCR.

Real-time PCR

RNA samples from each cell line were column-purified using the RNeasy Mini Kit (Qiagen, Hilden, Germany). Reverse transcription and real-time PCR (RT-PCR) were performed to determine the mRNA expression levels of *Cx26*, *Cx32*, *Cx43*, and *Cox-2*, using *GAPDH* as a

Table 1. Connexin43-specific siRNA oligonucleotide sequences.

Oligonucleotide name	Oligonucleotide sequences
**Connexin 43 Duplex Sequences**	5′-AGCGUUUGCUAUGACCAAUUCUUCC-3′<br>3′-UGUCGCAAACGAUACUGGUUAAGAAGG-5′
**Endogenous Gene Positive Control**	5′-GCCAGACUUUGUUGGAUUUGAAATT-3′<br>3′-AAUUUCAAAUCCAACAAAGUCUGGCUU-5′
**Negative Control**	5′-CGUUAAUCGCGUAUAAUACGCGUAT-3′<br>3′-AUACGCGUAUUAUACGCGAUUAACGAC-5′

https://doi.org/10.1371/journal.pntd.0006843.t001

Table 2. Oligonucleotide primers and detection probe for real-time PCR.

Oligonucleotide name	Oligonucleotide sequences
**Connexin 26**	
**GJB2 F**<br>**GJB2 R**<br>**GJB2 probe**	CCC CTA AAG CCT CAA AAC AAA G<br>GAA ACA AAT GCC GAT ATC CTC TG<br>56-FAM/CCT TAC ACC /ZEN/AAT AAC CCC TAA CAG CCT /3IABkFQ
**Connexin 32**	
**GJB1 F**<br>**GJB1 R**<br>**GJB1 probe**	GCA CAG ACA TGA GAC CAT AGG<br>CAA ACC TGT CCA GTT CAT CCT<br>56-FAM/CCT ATC CCT /ZEN/GAG GCC ACC CAG /3IABkFQ
**Connexin 43**	
**GJA1 F**<br>**GJA1 R**<br>GJA1 probe	ACT TGG CGT GAC TTC ACT AC<br>AGC AGT TGA GTA GGC TTG AAC<br>56-FAM/AGG CAA CAT /ZEN/GGG TGA CTG GAG C/3IABkFQ
**Cox-2**	
**PTGS2 F**<br>**PTGS2 R**<br>**PTGS2 probe**	ACT TGG CGT GAC TTC ACT AC<br>AGC AGT TGA GTA GGC TTG AAC<br>/56-FAM/AGG CAA CAT /ZEN/GGG TGA CTG GAG C/3IABkFQ/
**GAPDH**	
**GAPDH F**<br>**GAPDH R**<br>**GAPDH probe**	ACA TCG CTC AGA CAC CAT G<br>TGT AGT TGA GGT CAA TGA AGG G<br>5HEX/AAG GTC GGA /ZEN/GTC AAC GGA TTT GGT C/3IABkFQ

https://doi.org/10.1371/journal.pntd.0006843.t002

control (Applied Biosystems, Santa Clara, CA, USA). The levels of RNA expression were calculated using the 2ΔΔCt method and individual expression data was normalized to GAPDH. The thermal cycling parameters for reverse transcription were modified according to the Applied Biosystems manual. Hexamer incubation at 25˚C for 10 min and reverse transcription at 42˚C for 30 min was followed by reverse transcriptase inactivation at 95˚C for 5 min. cDNA (20 ng) from the previous step was subjected to qRT-PCR using specific sets of primers (Table 2 in a total reaction volume of 25 μL (Applied Biosystems). qRT-PCR was performed in an optical 96-well plate using an ABI PRISM 7900 HT Sequence Detection System (Applied Biosystems) and TaqMan probe detection chemistry. The running protocol was as follows: initial denaturation at 95˚C for 10 min, and 40 cycles of amplification at 95˚C for 15 s and 60˚C for 1 min. After PCR, a dissociation curve was constructed by increasing the temperature from 65˚C to 95˚C to evaluate the PCR amplification specificity. The cycle threshold (Ct) value was recorded for each sample.

Statistical analysis

Statistical significance was analyzed using Student's *t*-tests. Differences were considered statistically significant at a $^{*}P < 0.05$, $^{**}P < 0.01$ and $^{***}P < 0.001$ versus control. Data are presented as the mean ± standard error of the mean (SEM) from at least three independent experiments.

Results

ESP and NDMA synergistically increase H69 cell proliferation

The roles of NDMA and ESP in the proliferation of H69 cells, as determined by cell viability, were investigated. Proliferation was higher in H69 cells treated with NDMA and ESP of *C. sinensis* than in control cells. Compared to control cells, the average increase for cells treated

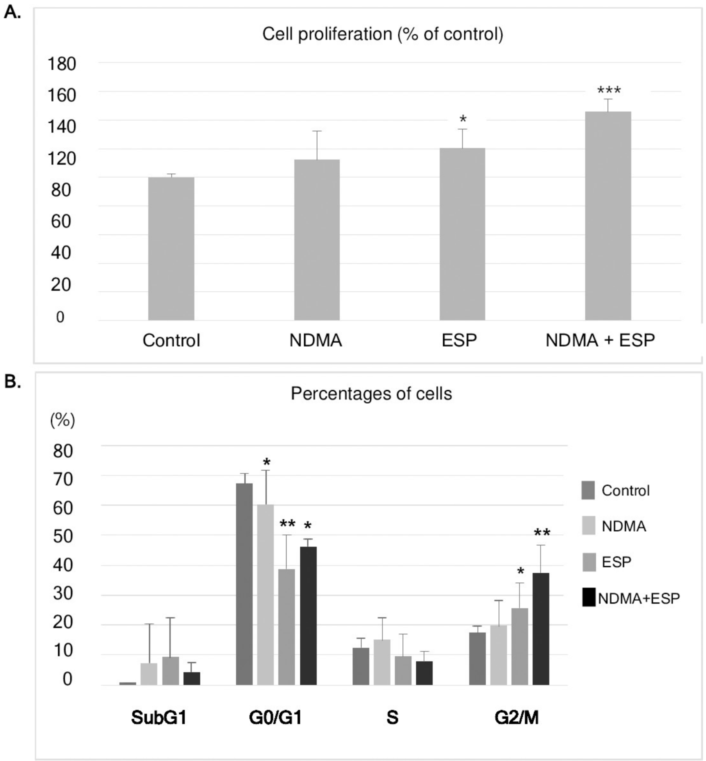

Fig 1. Effects of NDMA and ESP of *C. sinensis* on cell proliferation and cell cycle progression in human cholangiocytes (H69 cells). A. Cells were plated in 96-well plates (5×10^3 cells/well). Cell proliferation for each treatment was determined using the PrestoBlue cell viability assay. **B.** After H69 cells were treated with NDMA and ESP for 72 h, PI staining was performed to determine the percentage of cells in each phase. Data represent the mean ± SE of five independent experiments. $^{*}P < 0.05$, $^{**}P < 0.01$ and $^{***}P < 0.001$ versus control. (NDMA; N-nitrosodimethylamine, ESP; *Clonochis sinensis* excretory-secretory protein).

https://doi.org/10.1371/journal.pntd.0006843.g001

with NDMA was 112%, for cells treated with ESP was 120% ($P < 0.05$), and for cells treated with NDMA + ESP was 146% ($P < 0.001$). NDMA and ESP had synergistic effects on cell proliferation (Fig 1A).

Cell cycle distribution upon NDMA and/or ESP treatment in H69 cells

Cell cycle progression was monitored using propidium iodide (PI) staining (Fig 1B). In H69 cells treated with NDMA, ESP, or NDMA + ESP for 72 h, the number of cells in SubG1 phase did not change significantly, whereas cell numbers in the G0/G1 phase significantly decreased, but cells in the G2/M phase increased significantly compared to cell counts in the control group (Fig 1B). Fewer S-phase cells were identified in the ESP- and NDMA + ESP-treated cells than in control cells. There is no significant increase in G2/M phase in cells treated with NDMA compared to control group (Fig 1B). The proportions of G2/M-phase cells in each condition were as follows: control, 17%; NDMA, 20%; ESP, 26%; NDMA + ESP, 37% ($P = 0.007$).

Upregulation of cell proliferation—And cancer-related proteins by NDMA and ESP in H69 cells

Immunoblotting was utilized to detect the regulation of cell-cycle-related proteins in each group using calnexin as a loading control. The expression levels of several cell proliferation-

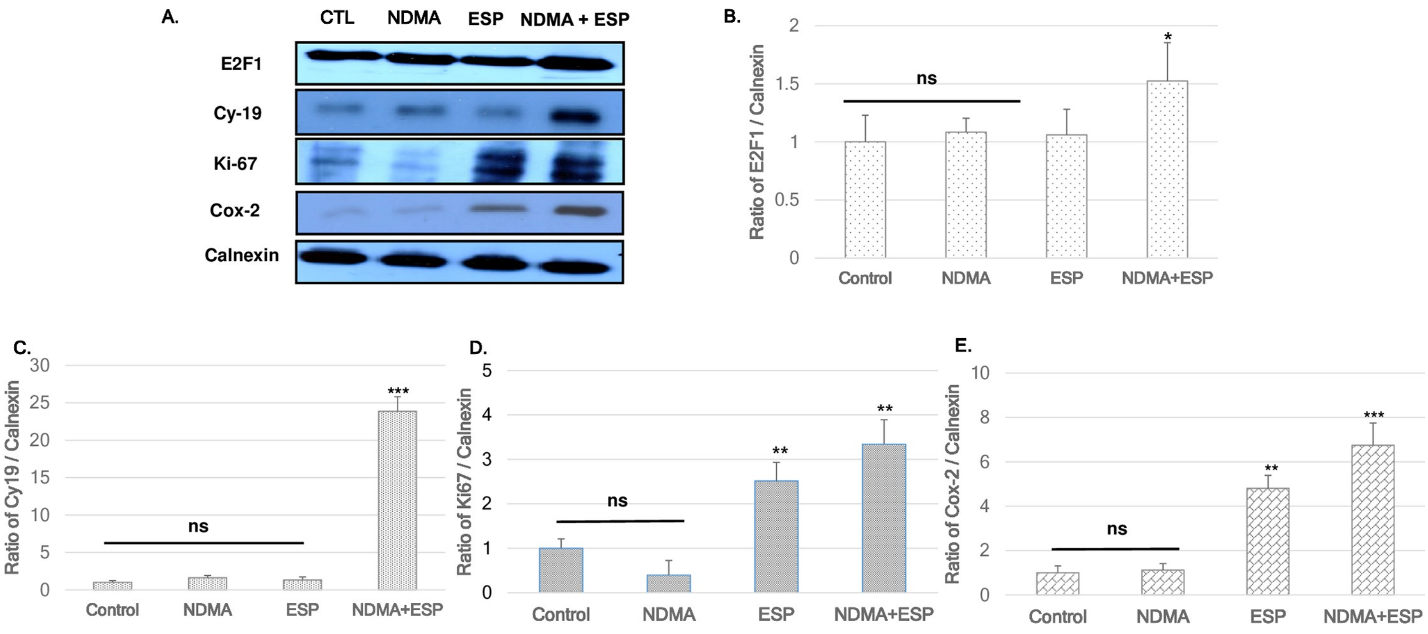

Fig 2. Expression of E2F1, Ki-67, Cy-19, and Cox-2 in H69 cells after treatment with NDMA and ESP, assessed by western blotting. H69 cells were incubated with PBS (vehicle), NDMA, ESP, or both for 72 h. The cells were collected for protein extraction. **A.** The blots of each groups were run under same experimental conditions and the images were cropped from different parts of the same gels. **B-E.** Quantification of relative E2F1, Ki-67, Cy-19, and Cox-2 expression in each group. The each proteins level are indicated as normalization of the ratio of E2F1/Calnexin, Ki-67/Calnexin, Cy-19/Calnexin, and Cox-2/Calnexin. DATA represent the mean ± SE of five independent experiments.$^{*}P < 0.05$, $^{**}P < 0.01$ and $^{***}P < 0.001$ versus control.

https://doi.org/10.1371/journal.pntd.0006843.g002

including E2F1, Ki-67 and Cy-19, and Cox-2 (an essential regulator of the G2/M transition) as cancer-related makers, were upregulated, especially in NDMA + ESP-treated cells (Fig 2).

Gap junction proteins in H69 cells

The expression levels of Cx26 and Cx43 were high in NDMA + ESP-treated cells, as confirmed by immunoblotting (Fig 3). The intracellular concentrations of Cx26 and Cx43 in each condition were observed under a confocal microscope. Western blotting indicated increases in Cx26 and Cx43 expression in cells treated with ESP and NDMA + ESP (Fig 3). The expression of Cx32 (blue) was markedly lower in cells treated with NDMA + ESP than in other cells. Immunofluorescence confocal microscopy indicated that the intracellular concentrations of Cx26 (green) and Cx43 (green) increased in cells treated with NDMA + ESP (Fig 4).

Reduced cell proliferation upon NDMA and ESP treatment in H69 cells by Cx43 silencing compare to silencing negative control

Downregulation of Cx43 by Cx43-specific small interfering RNA resulted in a slight increase in cell proliferation, but the differences among groups were not significant, even though ESP and NDMA synergistically stimulated cell proliferation in silencing negative control. The average increases compared to the control were as follows: NDMA, 104.5%; ESP, 105.1%; and NDMA + ESP, 107.6% (Fig 5A).

Downregulation of Cx43 by Cx43-specific small interfering RNA reduced the expression of Cx26 and Cox-2 in H69 cells

To evaluate the effect of Cx43 downregulation on the expression of other gap junction proteins and Cox-2, H69 cells were harvested after treatment with NDMA and ESP of *C. sinensis* for 72

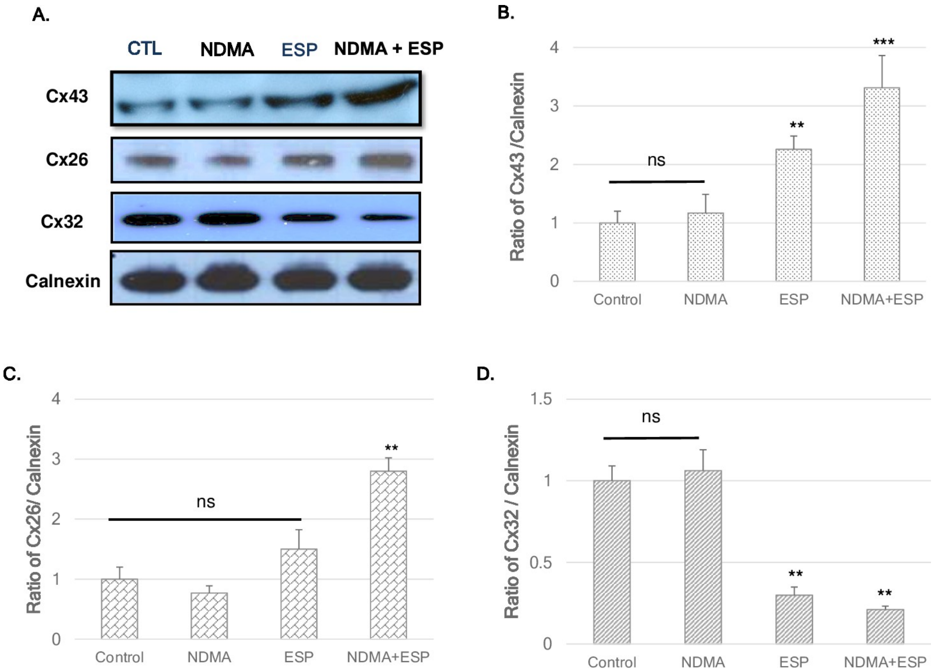

Fig 3. Expression of the gap-junction proteins connexin 26, connexin 32, and connexin 43 in H69 cells after treatment with NDMA and/or ESP, as determined by western blotting. H69 cells were incubated with either PBS (vehicle) or NDMA and/or ESP for 72 h, and the cells were collected for protein extraction. **A.** The blots of each groups were run under same experimental conditions and the images were cropped from different parts of the same gels. **B-D.** Quantification of relative Cx43 Cx26, and Cx32 expression in each group. The each proteins level are indicated as normalization of the ratio of Cx43/Calnexin, Cx26/Calnexin, and Cx32/Calnexin, and Cox-2/Calnexin. DATA represent the mean ± SE of five independent experiments. $^{*}P < 0.05$, $^{**}P < 0.01$ and $^{***}P < 0.001$ versus control.

https://doi.org/10.1371/journal.pntd.0006843.g003

h. Transfection with Cx43 siRNA resulted in a reduction in *Cx43* expression of greater than 70% when compared with that of the liposome-only control (Fig 5A). Real-time PCR showed that Cx43 silencing resulted in the downregulation of *Cx26* and *Cox-2* (Fig 5C and 5D, respectively). However, *Cx32* expression was not affected in H69 cells transfected with *Cx43*-specific siRNA.

Discussion

Our results provided the first evidence for the involvement of gap junction proteins in the pathogenesis of CHCA by *C*. *sinensis*. NDMA and ESP of *C*. *sinensis* increased Cx43 expression substantially in H69 human cholangiocytes. In addition, in the presence of ESP and NDMA stimulation, *Cx43* knockdown inhibited *Cox-2* expression in H69 cells. Yan et al. [15] reported that iNOS is highly expressed in Kupffer cells, sinusoidal endothelial cells, and biliary epithelial cells in BALB/c mice after *C*. *sinensis* infection. Nitric oxide (NO) formation and nitrosation may contribute to the development of *C*. *sinensis*-associated carcinogenesis [11, 12, 14, 15]. The induction of iNOS under inflammatory conditions suggests that NO is involved in the upregulation of *Cx43* [16, 19]. Therefore, we also expect the involvement of iNOS in the elevation of *Cx43* expression under inflammatory conditions in the present *C sinensis* model. Further studies are warranted to test this hypothesis.

Our results indicated that ESP of *C*. *sinensis* and NDMA had a synergistic effect on the proliferation of human cholangiocytes (Fig 1). In addition to the observed increase in cell proliferation and alteration of the cell cycle, the expression of the gap junction proteins Cx43 and

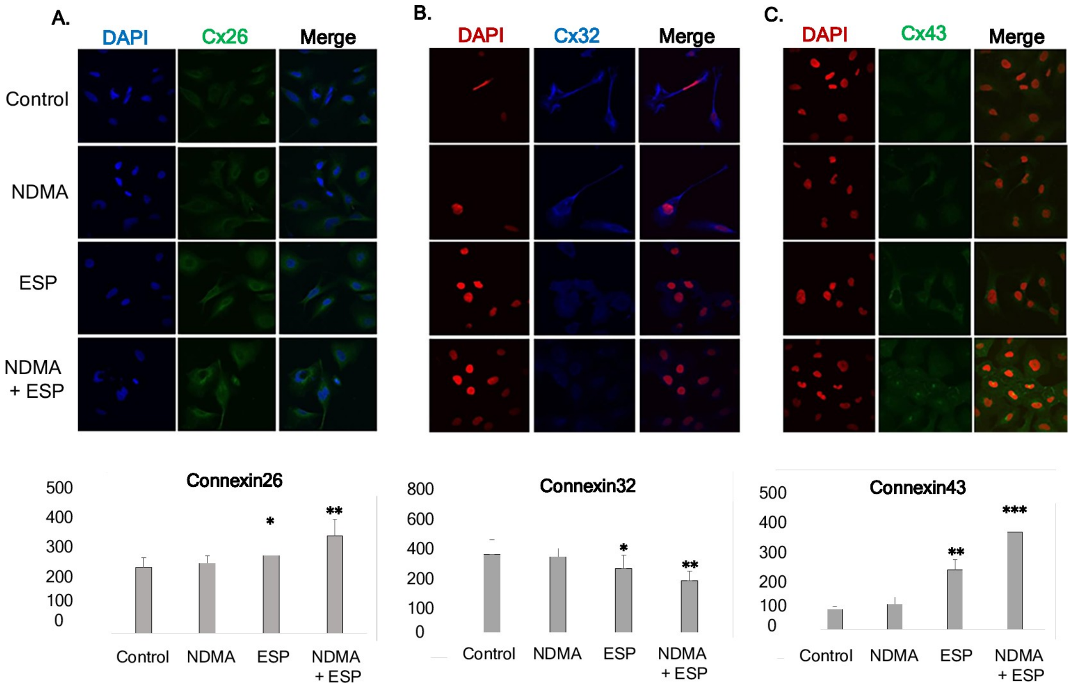

Fig 4. Concentration of intracellular connexin 26, connexin 32, and connexin 43 in human cholangiocytes (H69 cells). After treatment with NDMA and ESP for 72 h, the concentration of intracellular connexin 26, 32, and 43 in H69 cells was measured by laser scanning microscopy (×4,000). A-C: Immunofluorescence confocal microscopy indicated that the intracellular concentrations of Cx26 (green)/DAPI (blue), Cx32 (blue)/ DAPI (red), and Cx43 (green)/ DAPI (red) increased in cells treated with NDMA + ESP. Scale bar = 25 μm. Data represent the mean ± SE of five independent experiments. $^{*}P < 0.05$, $^{**}P < 0.01$ and $^{***}P < 0.001$ versus control.

https://doi.org/10.1371/journal.pntd.0006843.g004

Cx26 increased in H69 cells treated with NDMA and ESP. Most normal cells have functional gap junctional intercellular communication, in contrast to the dysfunctional communication of cancer cells [17–22]. When used in combination with NDMA, ESP of *C*. *sinensis* induced cell proliferation and increased the expression of E2F1, and Ki-67 as cell proliferation proteins. Additionally, as shown in Fig 1B, treatment with NDMA + ESP maximized the proportion of cells in the G2/M phase, implying that NDMA and ESP synergistically stimulate cell cycle progression. We analyzed the expression of cell proliferation-, cell cycle-, and cancer-related proteins (Fig 2), including E2F1, Ki-67, and Cy19, Cox-2 [24–26]. E2F1 is able to induce cell cycle progression, resulting in cell proliferation [3, 24]. Consistent with these previous findings, we observed that increased expression of E2F1 stimulated cell proliferation. C-Met is involved in early events of carcinogenesis, and Ki-67 is involved in the formation of invasive carcinoma [25–28]. Biliary epithelial cells retain Cy19 expression after neoplastic transformation in almost all cases [26, 28]. In our study, Cy19 and Ki-67 were upregulated in response to the stimulation of H69 cell proliferation.

Cyclooxygenase 2 (Cox-2), an enzyme involved in the production of prostaglandins, was over-expressed when cells were stimulated by NDMA and ESP. Cox-2 over-expression has been observed in various inflammatory diseases and in bile duct carcinoma cells, mainly in the cytoplasm [29–30]. Importantly, bile duct epithelial cells in primary sclerosing cholangitis show very strong Cox-2 expression, comparable to that in carcinoma cells. In contrast, epithelial cells in primary biliary cirrhosis show moderate levels of Cox-2 expression [29, 30]. In this context, the over-expression of Cy19, Ki-67, and Cox-2 may result in the transformation of normal H69 cells to cancer-like cells by stimulation with NDMA and ESP of *C*. *sinensis*. In the

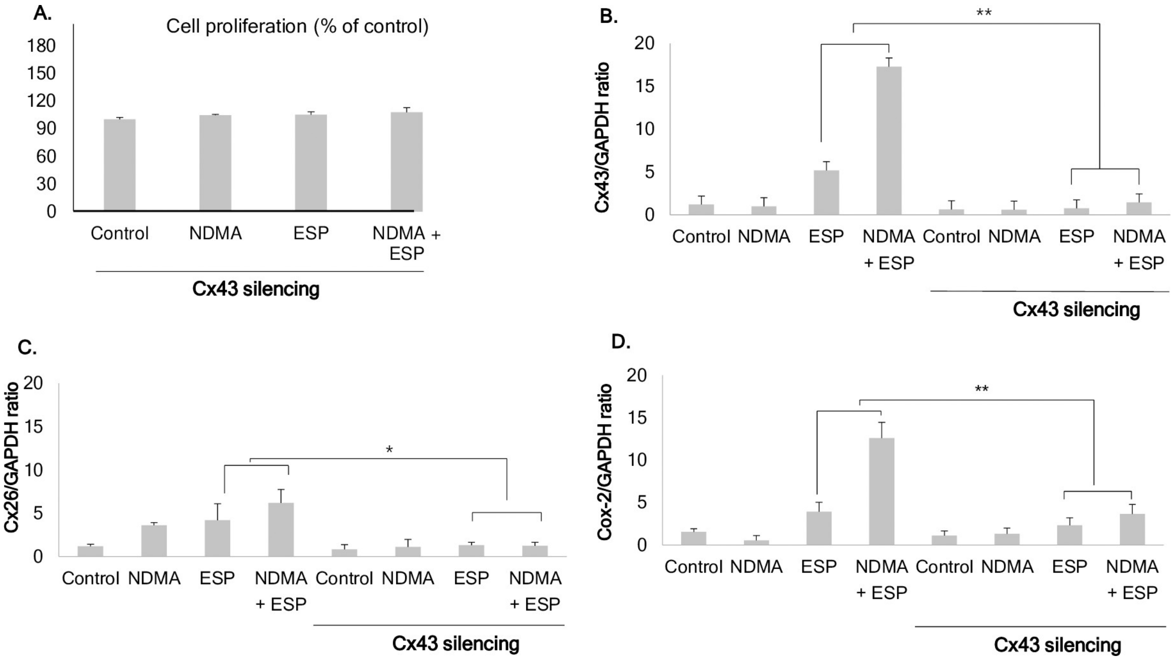

Fig 5. Effect of connexin 43 silencing in H69 cells. A. Reduced cell proliferation upon NDMA and ESP treatment in H69 cells by connexin 43 silencing. **B.** Uptake of Cx43 siRNA reduces *Cx43* expression, as confirmed by real-time PCR. *Cx43* expression was remarkably reduced in H69 cells transfected with *Cx43*-specific siRNA. **C.** Ratio of *Cx26/GAPDH* in H69 cells transfected with *Cx43*-specific siRNA. *Cx26* expression was remarkably reduced in H69 cells transfected with *Cx43*-specific siRNA. **D.** The ratio of *Cox-2/GAPDH* in H69 cells transfected with *Cx43*-specific siRNA. *Cox-2* expression was remarkably reduced in H69 cells transfected with *Cx43*-specific siRNA. Data represent the mean ± SE of five independent experiments. $^{*}P < 0.001$ versus control siRNA.

https://doi.org/10.1371/journal.pntd.0006843.g005

present study, Cx43 and Cx26 expression levels were increased in H69 cells upon stimulation with NDMA and ESP of *C. sinensis*. In contrast, Cx32 was significantly downregulated. Increased expression of hepatic Cx43 was noted in cirrhosis and in a mouse model of acute-on-chronic liver failure in response to LPS, and this effect was related to the severity of inflammation [19]. This increased Cx43 expression was likely an adaptive protective response of the liver to enable better cell-to-cell communication [16, 20, 21]. The expression levels of Cx26 and Cx32, major connexins in the liver, are extremely low in several HCC lines, but Cx43, a minor connexin in the liver, is highly expressed in metastatic cancer [17, 20–22]. Faniku et al. [30] reported that the inhibition of Cx43 signaling plays a more significant role in regulating

Table 3. Full spectrum of connexins expressed in rodent and human livers.

Connexins	Localization	References
**Cx26**	HP, KC, SC, SEC	[16], [20]
**Cx32**	HP, BEC, SEC	[21], [23]
**Cx37**	AEC, PEC	[18], [20]
**Cx39**	NS	[20]
**Cx40**	AEC, PEC	[17]
**Cx43**	AEC, BEC, GC, KC, PEC, SC, SEC	[16], [23], [30], [31], [32]

AEC, hepatic artery endothelial cell; BEC, biliary epithelial cell
GC, Glisson's capsule; HP, hepatocyte; KC, Kupffer cell; NS, not specified
PEC, portal vein endothelial cell; SC, stellate cell; SEC, sinusoidal endothelial cell.

https://doi.org/10.1371/journal.pntd.0006843.t003

cell proliferation than cell migration. Especially, the knockdown of Cx43 inhibited significantly the proliferation and migration such as vascular smooth muscle cells, epithelial cells in vitro [30–32]. Cx43 knockdown using siRNA reduced cell proliferation and significantly suppressed the expression of Cx26 and Cox-2 in H69 cells stimulated with NDMA and ESP compare to silencing negative control stimulated with NDMA and ESP. The connexin proteins Cx43 and Cx26 are involved in cell modification upon stimulation by NDMA and ESP of *C. sinensis*.

In general, cells contain several known connexins, classified according to their intracellular location (Table 3).

Intercellular communication via gap junctions is inhibited by increased Cox-2 expression, as is frequently observed in several forms of human malignancies [29, 33]. Recently, several reports have suggested that the carcinogenic mechanisms of hydrogen peroxide, TPA, and quinones may be involved in the inhibition of GJIC by Cx43 phosphorylation via ERK1/2 activation in rat liver epithelial cells [13, 17]. Furthermore, increased expression of Cx43 is positively correlated with NFκB activation in muscular arteries of patients undergoing coronary artery bypass graft surgery [34]. NFκB plays a central role in general inflammatory and immune responses. The 5′-flanking region of the *Cox-2* promoter contains an NFκB binding site, and NFκB is a critical regulator of Cox-2 expression in many cell lines [13, 34]. Taken together, the present findings suggest that Cx43 expression induces Cox2 over-expression via NFκB activation. However, until now, the link between NFκB activation, Cx43 expression, and Cox2 over-expression has not been clearly established. In the future, it will be interesting to examine the relationship between the GJIC and NFκB activation, Cox-2 by ESP of *C. sinensis* and NDMA.

In conclusion, our results suggest that Cx43 plays a key role in cell proliferation, potentially leading to CHCA development upon stimulation by ESP of *C. sinensis* and NDMA.

Acknowledgments

We thank Professor Dae-Gon Kim of Chonbuk National University for providing H69 cells. We would like to thank Professor Sung-Jong Hong and Fuhong Dai, Department of Medical Environmental Biology, Chung-Ang University College of Medicine, for providing adult worms of *C. sinensis*.

Author Contributions

Conceptualization: Eun-Min Kim.

Data curation: Eun-Min Kim, Sung-Tae Hong.

Formal analysis: Eun-Min Kim, Young Mee Bae, Min-Ho Choi.

Funding acquisition: Eun-Min Kim, Sung-Tae Hong.

Investigation: Eun-Min Kim, Young Mee Bae.

Methodology: Eun-Min Kim, Young Mee Bae.

Project administration: Eun-Min Kim, Young Mee Bae.

Resources: Eun-Min Kim.

Software: Eun-Min Kim.

Supervision: Eun-Min Kim, Sung-Tae Hong.

Validation: Eun-Min Kim, Young Mee Bae, Min-Ho Choi.

Visualization: Eun-Min Kim.

Writing – original draft: Eun-Min Kim, Sung-Tae Hong.

Writing – review & editing: Eun-Min Kim, Min-Ho Choi, Sung-Tae Hong.

References

1. Bouvard V, Barn R, Straif K, Grosse Y, Secretan B, El Ghissassi F, et al. A review of human carcinogens-Part B: biological agents. Lancet Oncol. 2009; 10: 321–322. PMID: 19350698
2. Kim EM, Kim JL, Choi SY, Kim JW, Kim S, Choi MH, et al. Infection status of freshwater fish with metacercariae of *Clonorchis sinensis* in Korea. Korean J Parasitol. 2008; 46: 247–251. https://doi.org/10.3347/kjp.2008.46.4.247 PMID: 19127331
3. Kim EM, Kim JS, Choi MH, Hong ST, Bae YM. Effects of excretory/secretory products from *Clonorchis sinensis* and the carcinogen dimethylnitrosamine on the proliferation and cell cycle modulation of human epithelial HEK293T cells. Korean J Parasitol. 2008; 46: 127–132. https://doi.org/10.3347/kjp.2008.46.3.127 PMID: 18830050
4. Kim EM, Bae YM, Choi MH, Hong ST. Cyst formation, increased anti-inflammatory cytokines and expression of chemokines support for *Clonorchis sinensis* infection in FVB mice. Parasitol Int. 2012; 61: 124–129. https://doi.org/10.1016/j.parint.2011.07.001 PMID: 21820080
5. Kim EM, Yu HS, Jin Y, Choi MH, Bae YM, Hong ST. Local immune response to primary infection and re-infection by *Clonorchis sinensis* in FVB mice. Parasitol Int. 2017; 66: 436–442. https://doi.org/10.1016/j.parint.2016.11.006 PMID: 27856336
6. Kim EM, Kwak YS, Yi MH, Kim JY, Sohn WM, Yong TS. *Clonorchis sinensis* antigens alter hepatic macrophage polarization in vitro and in vivo. PLoS Negl Trop Dis. 2017; 24: 11.
7. Hong ST, Fang Y. *Clonorchis sinensis* and clonorchiasis, an update. Parasitol Int. 2012; 61: 17–24. https://doi.org/10.1016/j.parint.2011.06.007 PMID: 21741496
8. Pinlaor S, Hiraku Y, Ma N, Yongvanit P, Semba R, Oikawa S, et al. Mechanism of NO-mediated oxidative and nitrative DNA damage in hamsters infected with *Opisthorchis viverrini*: a model of inflammation-mediated carcinogenesis. Nitric Oxide. 2004; 11: 175–183. https://doi.org/10.1016/j.niox.2004.08.004 PMID: 15491850
9. Yongvanit P, Pinlaor S, Bartsch H. Oxidative and nitrative DNA damage: key events in opisthorchiasis-induced carcinogenesis. Parasitol Int. 2012; 61: 130–135. https://doi.org/10.1016/j.parint.2011.06.011 PMID: 21704729
10. George J, Murray M, Byth K, Farrell GC. Differential alterations of cytochrome P450 proteins in livers from patients with severe chronic liver disease. Hepatology. 1995; 2: 120–128.
11. Lee JH, Rim HJ, Bak UB. Effect of *Clonorchis sinensis* infection and dimethylnitrosamine administration on the induction of cholangiocarcinoma in Syrian golden hamsters. Korean J Parasitol. 1993; 31: 21–30. PMID: 8390293
12. Uddin MH, Choi MH, Kim WH, Jang JJ, Hong ST. Involvement of PSMD10, CDK4, and tumor suppressors in development of intrahepatic cholangiocarcinoma of syrian golden hamsters induced by *Clonorchis sinensis* and *N*-nitrosodimethylamine. PLoS Negl Trop Dis. 2015; 9: e000400.
13. Surh YJ, Lee JY, Choi KJ, Ko SR. Effects of selected ginsenosides on phorbol ester-induced expression of cyclooxygenase-2 and activation of NF-kappaB and ERK1/2 in mouse skin. Ann N Y Acad Sci. 2002; 973: 396–401. PMID: 12485900
14. Yan C, Wang YH, Yu Q, Cheng XD, Zhang BB, Li B, et al. *Clonorchis sinensis* excretory/secretory products promote the secretion of TNF-alpha in the mouse intrahepatic biliary epithelial cells via Toll-like receptor 4. Parasit Vectors. 2015; 8: 559. https://doi.org/10.1186/s13071-015-1171-0 PMID: 26497121
15. Yan C, Li B, Fan F, Du Y, Ma R, Cheng XD, et al. The roles of Toll-like receptor 4 in the pathogenesis of pathogen-associated biliary fibrosis caused by *Clonorchis sinensis*. Sci Rep. 2017; 7: 3909. https://doi.org/10.1038/s41598-017-04018-8 PMID: 28634394
16. Balasubramaniyan V, Dhar DK, Warner AE, Vivien Li WY, Amiri AF, Bright B, et al. Importance of Connexin-43 based gap junction in cirrhosis and acute-on-chronic liver failure. J Hepatol. 2013; 58: 1194–1200. https://doi.org/10.1016/j.jhep.2013.01.023 PMID: 23376361
17. Chaytor AT, Martin PE, Edwards DH, Griffith TM. Gap junctional communication underpins EDHF-type relaxations evoked by ACh in the rat hepatic artery. Am. J Physiol Heart Circ Physiol. 2001; 280: H2441–H2450. https://doi.org/10.1152/ajpheart.2001.280.6.H2441 PMID: 11356596
18. Saito T, Krutovskikh V, Marion MJ, Ishak KG, Bennett WP, Yamaskai H. Human hemangiosarcomas have a common polymorphism but no mutations in the connexin37 gene. Int J Cancer. 2000; 86: 67–70. PMID: 10728596

19. Li K, Yao J, Shi L, Sawada N, Chi Y, Yan Q, et al. Reciprocal regulation between proinflammatory cytokine-induced inducible NO synthase (iNOS) and connexin43 in bladder smooth muscle cells. J Biol Chem. 2011; 286: 41552–41562. https://doi.org/10.1074/jbc.M111.274449 PMID: 21965676

20. Vinken M, De Kock J, Oliveira AG, Menezes GB, Cogliati B, Dagli ML, et al. Modifications in connexin expression in liver development and cancer. Cell Commun Adhes. 2012; 19: 55–62. https://doi.org/10.3109/15419061.2012.712576 PMID: 22950570

21. Bode HP, Wang L, Cassio D, Leite MF, St-Pierre MV, Hirata K, et al. Expression and regulation of gap junctions in rat cholangiocytes. Hepatology. 2002; 36: 631–640. https://doi.org/10.1053/jhep.2002.35274 PMID: 12198655

22. Villares GJ, Dobroff AS, Wang H, Zigler M, Melnikova VO, Huang L, et al. Overexpression of protease-activated receptor-1 contributes to melanoma metastasis via regulation of connexin 43. Cancer Res. 2009; 15: 6730–6737.

23. Fujimoto E, Sato H, Nagashima Y, Negishi E, Shirai S, Fukumoto K, et al. A Src family inhibitor (PP1) potentiates tumor-suppressive effect of connexin 32 gene in renal cancer cells. Life Sci. 2005; 76: 2711–2720. https://doi.org/10.1016/j.lfs.2004.10.049 PMID: 15792837

24. Zhu W, Giangrande PH, Nevins JR. E2Fs link the control of G1/S and G2/M transcription. EMBO J. 2004; 23: 4615–4626. https://doi.org/10.1038/sj.emboj.7600459 PMID: 15510213

25. Sanada Y, Osada S, Tokuyama Y, Tanaka Y, Takahashi T, Yamaguchi K, et al. Critical role of c-Met and Ki-67 in progress of biliary carcinoma. Am Surg. 2010; 76: 372–379. PMID: 20420246

26. Chatzipantelis P, Lazaris AC, Kafiri G, Papadimitriou K, Papathomas TG, Nonni A, et al. Cytokeratin-7, cytokeratin-19, and c-Kit: Immunoreaction during the evolution stages of primary biliary cirrhosis. Hepatol Res. 2006; 363: 182–187.

27. Tan XP, Zhang Q, Dong WG, Lei XW, Yang ZR. Upregulated expression of Mina53 in cholangiocarcinoma and its clinical significance. Oncol Lett. 2012; 3: 1037–1041. https://doi.org/10.3892/ol.2012.620 PMID: 22783387

28. Aishima S, Nishihara Y, Kuroda Y, Taguchi K, Iguchi T, Taketomi A, et al. Histologic characteristics and prognostic significance in small hepatocellular carcinoma with biliary differentiation: subdivision and comparison with ordinary hepatocellular carcinoma. Am J Surg Pathol. 2007; 31: 783–791. https://doi.org/10.1097/01.pas.0000213421.53750.0a PMID: 17460464

29. Hayashi N, Yamamoto H, Hiraoka N, Dono K, Ito Y, Okami J, et al. Differential expression of cyclooxygenase-2 (COX-2) in human bile duct epithelial cells and bile duct neoplasm. Hepatology. 2001; 34: 638–650. https://doi.org/10.1053/jhep.2001.28198 PMID: 11584358

30. Faniku C, O'Shaughnessy E, Lorraine C, Johnstone SR, Graham A, Greenhough S, Martin PEM, et al. The Connexin Mimetic Peptide Gap27 and Cx43-Knockdown Reveal Differential Roles for Connexin43 in Wound Closure Events in Skin Model Systems. Int J Mol Sci. 2018; 18; 19(2): 604.

31. Paw M, Borek I, Wnuk D, Ryszawy D, Piwowarczyk K, Kmiotek K, Wójcik-Pszczoła KA, Pierzchalska M, Madeja Z, Sanak M, Błyszczuk P, Michalik M, Czyż J. Connexin43 Controls the Myofibroblastic Differentiation of Bronchial Fibroblasts from Patients with Asthma. Am J Respir Cell Mol Biol. 2017; 57:100–110. https://doi.org/10.1165/rcmb.2015-0255OC PMID: 28245135

32. Paw M, Wnuk D, Kądziołka D, Sęk A, Lasota S, Czyż J, Madeja Z, Michalik M. Fenofibrate Reduces the Asthma-Related Fibroblast-To-Myofibroblast Transition by TGF-B/Smad2/3 Signaling Attenuation and Connexin 43-Dependent Phenotype Destabilization. Int J Mol Sci. 2018;29: 19(9).

33. Lee KW, Chun KS, Lee JS, Kang KS, Surh YJ, Lee HJ. Inhibition of cyclooxygenase-2 expression and restoration of gap junction intercellular communication in H-ras-transformed rat liver epithelial cells by caffeic acid phenethyl ester. Ann N Y Acad Sci. 2004; 1030: 501–507. https://doi.org/10.1196/annals.1329.062 PMID: 15659835

34. Li JY, Lai YJ, Yeh HI, Chen CL, Sun S, Wu SJ, et al. Atrial gap junctions, NF-kappaB and fibrosis in patients undergoing coronary artery bypass surgery: the relationship with postoperative atrial fibrillation. Cardiology. 2009; 112: 81–88. https://doi.org/10.1159/000141012 PMID: 18580064
